# Substance use services for refugees

**DOI:** 10.2471/BLT.18.225086

**Published:** 2019-04-01

**Authors:** M Claire Greene, Peter Ventevogel, Jeremy C Kane

**Affiliations:** a Johns Hopkins Bloomberg School of Public Health, John Hopkins University, 615 N Wolfe St, Baltimore, MD 21205, United States of America.; b Public Health Section, United Nations High Commissioner for Refugees, Geneva, Switzerland.

In 2017, a record number of 68.5 million people were living in forced displacement. Over one-third of these are refugees who crossed country borders in search of safety and protection, primarily into low- and middle-income countries.^1^ Becoming a refugee influences risks for substance use disorders due to high levels of distress and mental health problems, disruption of protective community networks, transformation of social roles, changes in access to substances, and weakened enforcement of substance control policies.^2^ Epidemiological evidence corroborates the burden of substance use in forcibly-displaced populations, particularly among men and individuals with mental disorders.^3^^–^^5^

Addressing substance use disorders in refugee contexts falls within the mandates of various United Nations (UN) agencies. A new impetus for collaborative action is provided by the Global Compact on Refugees, which was recently adopted at the 2018 United Nations General Assembly and calls for joint action of multiple stakeholders to work together towards refugee protection, assistance and solutions.^6^

Guidelines for the provision of humanitarian assistance recommend actions to address substance misuse,^7^^,^^8^ but in practice few steps are being taken to monitor, prevent and treat substance use problems among displaced populations.^9^ A major reason for inaction is that available evidence on the effectiveness and implementation of substance use interventions in humanitarian settings is limited.^9^

The UN High Commissioner for Refugees commissioned a review to synthesize evidence on interventions for substance use among refugees in low- and middle-income countries and to identify promising strategies to inform operational research and pilot programming.^10^ The review identified six interventions evaluated among refugees and 29 interventions among other disadvantaged populations. Half of the refugee studies described screening and brief interventions to reduce hazardous substance use. Brief interventions are intended to prevent transition from hazardous use to disorder, while it is recommended that individuals who meet criteria for severe substance use disorder also receive more intensive psychosocial and/or pharmacological intervention. Harm reduction was described in one refugee study, yet the evaluation focused on implementation challenges, not effectiveness. The review did not identify studies describing promotion, universal prevention or selective prevention interventions in refugee populations.

Treatment studies identified in the review were not fully described or rigorously evaluated and had methodological limitations including non-experimental designs, non-validated measures, heterogeneous outcomes and inconsistent recall periods. Particularly striking was the lack of studies on substances other than alcohol and on treatment options for people with severe substance use disorder.

We argue that the gaps in knowledge and implementation should be addressed by operational research on substance use interventions among refugees. Such research should evaluate packages of interventions that bring together different approaches within a coherent frame. There will not be a single solution for addressing the complex public health problem of substance use disorder in refugee settings. Rather, various approaches that are known to work in non-refugee contexts should be implemented and studied in refugee settings while considering contextual aspects. Such approaches include the role of gender, health and social problems, and structural factors, for example the criminalization of illicit drug use. Such a comprehensive package will likely consist of community-based approaches to raise awareness, promote primary prevention and reduce substance-related harm. Brief interventions delivered within an integrated primary health-care model to identify, manage, treat and appropriately refer (when possible) people with substance misuse, many of whom may be seeking care for health problems exacerbated by substance use, will also be needed. Capacity building including supervision for non-specialist providers is essential to ensure that interventions are implemented with fidelity and providers are confident in their ability to deliver this type of care. Last, clinical interventions for individuals with more severe substance use disorders should be provided to enable these individuals to receive higher levels of care, including more intensive psychological interventions, medication-assisted treatment and medically assisted withdrawal management.

With the forcibly-displaced population growing, and with the increase in the burden of substance use disorder globally,^11^ substance use among refugees must be considered a public health priority and addressed through concerted actions. Humanitarian nongovernmental organizations often have insufficient research capacity and need to collaborate with research institutions and UN agencies. The limited available literature demonstrates that it is feasible to implement and evaluate substance use interventions in refugee populations. However, significant work needs to be done in overcoming implementation challenges and moving towards identifying evidence-based substance use services for refugees living in complex, rapidly changing contexts.^12^
